# Contribution of complement activation pathways to neuropathology differs among mouse models of Alzheimer's disease

**DOI:** 10.1186/1742-2094-8-4

**Published:** 2011-01-15

**Authors:** Maria I Fonseca, Shu-Hui Chu, Alisia M Berci, Marie E Benoit, Douglas G Peters, Yuko Kimura, Andrea J Tenner

**Affiliations:** 1 Department of Molecular Biology and Biochemistry, University of California, Irvine, Irvine, California, USA; 2 Department of Neurobiology and Behavior, University of California, Irvine, Irvine, California, USA; 3 Institute for Translational Medicine and Therapeutics, University of Pennsylvania School of Medicine, Philadelphia, PA 19104, USA; 4 Institute for Memory Impairments and Neurological Disorders, University of California, Irvine, Irvine, California, 92697, USA

## Abstract

**Background:**

Complement proteins and activation products have been found associated with neuropathology in Alzheimer's disease (AD). Recently, a C5a receptor antagonist was shown to suppress neuropathology in two murine models of AD, Tg2576 and 3xTg. Previously, a genetic deficiency of C1q in the Tg2576 mouse model showed an accumulation of fibrillar plaques similar to the complement sufficient Tg2576, but reactive glia were significantly decreased and neuronal integrity was improved suggesting detrimental consequences for complement activation in AD. The goal of this study was to define the role of the classical complement activation pathway in the progression of pathology in the 3xTg mouse that develops tangles in addition to fibrillar plaques (more closely reflecting human AD pathology) and to assess the influence of complement in a model of AD with a higher level of complement hemolytic activity.

**Methods:**

3xTg mice deficient in C1q (3xTgQ-/-) were generated, and both 3xTg and 3xTgQ-/- were backcrossed to the BUB mouse strain which has higher in vitro hemolytic complement activity. Mice were aged and perfused, and brain sections stained for pathological markers or analyzed for proinflammatory marker expression.

**Results:**

3xTgQ-/- mice showed similar amounts of fibrillar amyloid, reactive glia and hyperphosphorylated tau as the C1q-sufficient 3xTg at the ages analyzed. However, 3xTg and 3xTgQ-/- on the BUB background developed pathology earlier than on the original 3xTg background, although the presence of C1q had no effect on neuropathological and pro-inflammatory markers. In contrast to that seen in other transgenic models of AD, C1q, C4 and C3 immunoreactivity was undetectable on the plaques of 3xTg in any background, although C3 was associated with reactive astrocytes surrounding the plaques. Importantly, properdin a component of the alternative complement pathway was associated with plaques in all models.

**Conclusions:**

In contrast to previously investigated transgenic models of AD, development of neuropathology in 3xTg mice, which progresses much slower than other murine models, may not be influenced by fibrillar amyloid mediated activation of the classical complement pathway, suggesting that the alternative complement pathway activation or a C3-independent cleavage of C5 could account for the detrimental effects in these mice that are prevented by the C5a receptor antagonist. Furthermore, the paucity of complement activation may be a factor in the slower kinetics of progression of pathology in the 3xTg model of this disease.

## Background

Alzheimer's disease is a progressive neurodegenerative dementia of the elderly characterized by a well defined pathology that includes accumulation of β-amyloid in plaques, hyperphosphorylated tau that ultimately forms neurofibrillary tangles, and neuronal loss [1]. In addition to these hallmarks, a prominent inflammatory reaction, characterized by the presence of reactive glia associated with the fibrillar plaques, upregulation of several complement proteins [2-5] including local synthesis of the components [6,7] is observed. C1q is associated with fibrillar plaques as well as tangles [3,8], and the presence of C5b-9 associated with dystrophic neurites in plaques and with tangles [9] indicates that complement is fully activated in AD [10]. These *in vivo *observations, supported by the *in vitro *studies demonstrating that fibrillar β-amyloid can activate the classical [11,12] and alternative [13] complement pathways and that the complement activation fragment C5a is chemotactic for microglia [14], led to the hypothesis that the complement activation triggered by fibrillar ß-amyloid contributes to the inflammatory reaction that can play a detrimental role in the progression of the later stages of Alzheimer's disease [15].

Both a genetic and a pharmacological approach have been used to investigate this hypothesis. First, a Tg2576 transgenic mouse model of AD was crossed to a C1q-/- mouse to generate the APPQ-/- mouse which lacks C1q (the first component of the classical complement pathways). We observed a decrease in reactive glia associated with fibrillar amyloid plaques in the APPQ-/- compared to the APP mice at all ages analyzed. In addition, the APPQ-/- mice showed greater synaptophysin (SYN) and MAP-2 staining relative to the APP mice indicating a preservation of neuronal integrity [16]. In a second approach, Tg2576 mice were treated with a specific antagonist for CD88, a receptor for the complement activation fragment C5a, for three months. The treated animals showed a decrease in plaque and glia pathology, an increase in the SYN staining and cognitive improvement [17]. 3xTg mice, a mouse model of AD that develops neurofibrillar tangles as well as plaques, similarly treated also showed a decrease in plaques, reactive glia and, in addition, a decrease in hyperphosphoryated tau [18]. These results support the hypothesis that complement activation plays a detrimental role in AD since inhibiting classical complement activation or blocking the downstream pathway by inhibiting C5a/C5aR interaction renders a substantial improvement in pathology and behavior of these animals.

Since it has also been reported that C1q can bind to hyperphosphorylated tau and activate complement *in vitro *[8], the contribution of complement activation on the kinetics of appearance and accumulation of both amyloid plaques and phosphorylated tau, was assessed in the 3xTg and in the 3xTg lacking C1q (3xTgQ-/-) at different ages. In addition, a caveat for the use of standard mouse models for studying the involvement of complement in human AD is the reported weak hemolytic activity of mouse complement [19]. While the basis for this apparent deficiency seen in *in vitro *assays has not been delineated, one possible consequence *in vivo *would be a lower or less efficient C5 cleavage and thus a lower generation of both C5a, a proinflammatory peptide, and the membrane attack complex composed of C5b, C6, C7, C8 and C9 (C5b-9). This would result not only in a decrease in bystander damage of cells in the mouse system, but also decreased proinflammatory activity, relative to that in the human system. The BUB/BnJ strain of mice has higher complement hemolytic activity, as measured *in vitro*, than that of current transgenic mouse AD models [15,20-22]. To determine if this parameter translates into differences in pathology in the 3xTg model, the 3xTg was backcrossed (N = 6) to the BUB strain and to BUBQ-/-, and the development of pathology compared with that of the 3xTg on a mixed C57BL/6 background.

The results show that the presence or absence of C1q generated no difference in plaque or tangle pathology or inflammatory response in this 3xTg animal model. Interestingly however, C1q and C4 were not detected associated with the plaques in the 3xTg models, in contrast to prominent deposition in other transgenic models (Tg2576 and Arc48). These data indicate that the classical complement pathway may not contribute to the generation of C5a and inflammation in the 3xTg model which develops pathology more slowly than other models of this disease. However, an increase in pathology was observed in the 3xTg backcrossed to BUB background relative to the initial 3xTg. Since we previously demonstrated a suppression of pathology in this model with a C5a receptor antagonist, these results suggest that activation of the alternative complement pathway or the presence of other C5 cleaving enzymes may be the mechanism by which the proinflammatory peptide C5a is generated in this model.

## Methods

### Transgenic mice

3xTg mice harboring the Swedish mutation (KM670/671NL), a human four repeat Tau (P301L) mutation and a knock in mutation of presenilin1 (PS1M146V) [18] in a mixed background (3xTg) were generously provided by Dr. Frank LaFerla (UCI, Irvine, CA). These mice were backcrossed for 6 generations to the BUB/BnJ strain (The Jackson Laboratory, Bar Harbor, Maine) to generate 3xTgBUB mice. The 3xTg and 3xTgBUB were crossed to C1q knockout mice (C1qa-/-) [23] previously backcrossed onto C57BL/6 or onto the BUB background until homozygous for all markers (validated by PCR and/or QPCR and test breeding) generating 3xTgQ-/- and 3xTgQ-/-BUB respectively. The 3xTg colony generated from the initial 3xTg breeders has all the pathological features originally reported [18] but shows a slower progression of the AD pathology than the originally reported colonies and shows a significant gender difference in the pathology, as seen by some other investigators [24]. Since in our colony the females present significantly higher pathology than the males at all the ages, the data reported are from females only. Non transgenic mice or littermates of the same background were used as controls. Tg2576 [25] were maintained by breeding to B6/SJL obtained from Jackson Laboratory. Arc48, Arctic-mutant hAPP mice [26] (obtained from Dr. Lennart Mucke, Gladstone Institute, San Francisco, CA), were backcrossed onto the C57BL/6J strain (Jackson Laboratory).

### Tissue preparation

Mice were anesthetized with a mixture of ketamine/xylazine (67/27 mg/kg) and perfused with PBS. After dissection, half brain was immediately frozen on dry ice and the other half fixed overnight with 4% paraformaldehyde (for immunohistochemistry). Thereafter, fixed tissue was stored in PBS/0.02% Na azide at 4°C until use.

### Immunohistochemistry and image analysis

Vibratome sections (coronal, 40 um) were incubated sequentially with 3%H2O2/10%MeOH/TBS to block endoperoxidase, with 2% BSA/0.1%Triton/TBS to block non specific binding and with the corresponding primary antibodies or control IgG in blocking solution all as previously described [16]. Primary antibodies were detected with biotinylated secondary antibodies against the corresponding species, followed by ABC complex and DAB (VECTOR, Burlingame, CA). For immunofluorescence staining detection was done with species specific biotinylated antibodies followed by Cy3-Streptavidin (Jackson, West Grove, Pennsylvania, 1:200 dilution) or Alexa555-Streptavidin (SA) (Invitrogen, Carlsbad, CA, 1:200 dilution). For colocalization of C3 and GFAP, tissue was incubated with both primary antibodies simultaneously and C3 was detected with biotinylated anti rat antibody followed by Alexa555-SA, and GFAP was labeled with Alexa488 anti rabbit IgG. For immunofluorescence staining using anti C3d and C3b/iC3b/C3c antibodies primary antibodies were detected by incubation with the corresponding biotinylated secondary antibodies, followed by ABC complex. A final step with CY3-Tyramide (NEL 744, TSA plus, Perkin Elmer, Shelton CT) 1:50 dilution in amplification buffer was done as per manufacturer instructions. For single and double labeling, controls with the omission of the primary antibodies or the inclusion of normal IgG were performed and were negative. Primary antibodies used were: rabbit polyclonal anti mouse C1q (4 ug/ml, #1151 [27]) rat monoclonal anti CD45 (1 ug/ml) (Serotec, Raleigh, NC), mouse monoclonal anti hyperphosphorylated tau (AT8 or AT100, 0.2 and 0.02ug/ml respectively) (Pierce, Rockford, Il), rat monoclonal anti mouse C4 (clone16D2, 5ug/ml) (Cell Sciences) and rabbit anti mouse properdin antibody (5ug/ml) [28]. Three antibodies were used to detect C3: rat monoclonal anti mouse C3 (clone 11H9, 4ug/ml) (Cell Sciences, Canton, MA), rabbit polyclonal anti human C3c (10ug/ml) (Dako, Carpinteria, CA), and rabbit polyclonal anti human C3d (Dako, 0.4ug/ml). Rat monoclonal anti mouse C3b/iC3b/C3c (Cell Sciences, clone 2/11, 5ug/ml) specifically recognizes cleaved and not native C3. Fibrillar Aß was stained with 1% thioflavine as previously described [29].

Immunostaining was observed under a Zeiss Axiovert-200 inverted microscope (Carl Zeiss, Thornwood, NY) and images were acquired with a Zeiss Axiocam high-resolution digital color camera (1300x1030 pixel) using Axiovision 4.1 or 4.6 software. The same software (Carl Zeiss) was used to analyze the digital images. Percent of immunopositive area (% Field Area) (immunopositive area/total image area × 100) was determined for all the markers studied by averaging images of the subiculum area from 2-3 sections per animal. Digital images were obtained using the same settings and the segmentation parameters constant within a range per given marker and experiment. The mean value of the % Field Area for each marker in each animal was averaged per genotype group with the number of animals per group indicated in Figure legends. Data was analyzed using single ANOVA statistical analysis.

Staining of all animals from the same trial that were compared by image analysis was done simultaneously per given marker.

### Total RNA extraction, reverse transcription and quantitative real-time PCR (qRT-PCR)

Total RNA from pulverized frozen cortices (10 mg) and hippocampi (5 mg) was extracted using the Illustra RNAspin mini kit (GE Healthcare Life Sciences, Piscataway, NJ) following manufacturer's instructions. The cDNA synthesis was carried out with 100 ng of total RNA, 0.5 μg oligo(dT) primer, 40 units RNaseOUT and 200 units M-MLV reverse transcriptase RT (Invitrogen, Carlsbad, CA) according to the manufacturer's protocol. Quantitative PCR was performed using the iCycler iQ and the iQ5 software (Bio-Rad, Hercules, CA). Briefly, amplification was conducted in a 25 μl volume using 12.5 μl Maxima SYBR/Green Master Mix (Fermentas, Glen Burnie, MD), 100 ng of template cDNA and 0.3 μM each of forward and reverse gene-specific primers. The primers (IL-1α-F: AGACCGACCTCATTTTCTTCTG, IL-1α-R: ACCCGACTTTGTTCTTTGGTG, IL-1β-F: TACATCAGCACCTCACAAGCA, IL-1β-R: AGAAACAGTCCAGCCCATACT, CCL2-F: CACTCACCTGCTGCTACTCATTC, CCL2-R: CCATTCCTTCTTGGGGTCA, IL-6-F: AGGAGACTTCACAGAGGATACCA, IL-6-R: CATTTCCACGATTTCCCAGAG, TNFα-F: GGTGTTCATCCATTCTCTACC, TNFα-R: GAGCCATAATCCCCTTTCTAA, GAPDH-F: AACTCCCACTCTTCCACCTTC and GAPDH-R: GGTCCAGGGTTTCTTACTCCTT) were designed using the primer3 tool (http://frodo.wi.mit.edu/primer3/) and obtained from Eurofins MWG Operon (Huntsville, AL). RT was omitted in negative controls. The fold-difference in target genes cDNA relative to the GAPDH endogenous control was determined using the relative quantification method as follows: Fold-difference = 2^-ΔCt^, ΔCt = (Ct_Target _- Ct_GAPDH_) where Ct values are defined as the number of cycles for which the fluorescence signals were detected [30]. Results are represented as individual scatter dot plot with mean ± SEM of fold-difference (2^-ΔCt^) for each genotype group and compared with two-tailed t-test (Bonferroni post hoc test, alpha error = 0.05) and Pearson rank correlation coefficient. Differences were considered significant when p was <0.05.

## Results

### 3xTg and 3xTgQ-/- show similar pathology at all the ages tested, with trends for accelerated pathology in the 3xTg on the BUB background

The kinetics of progression of plaque deposition, activated glia and tangle pathology in the 3xTg as compared to the 3xTg backcrossed to a C1q knock out, 3xTgQ-/-, was assessed on animals from 7 months (m) to 18 m of age to permit comparison at multiple stages of plaque formation. In our colony of 3xTg mice, there is no plaque deposition seen in 7 m animals, while by 18 m there are a significant number of plaques in the subiculum area of the hippocampus.

**Figure 1:**
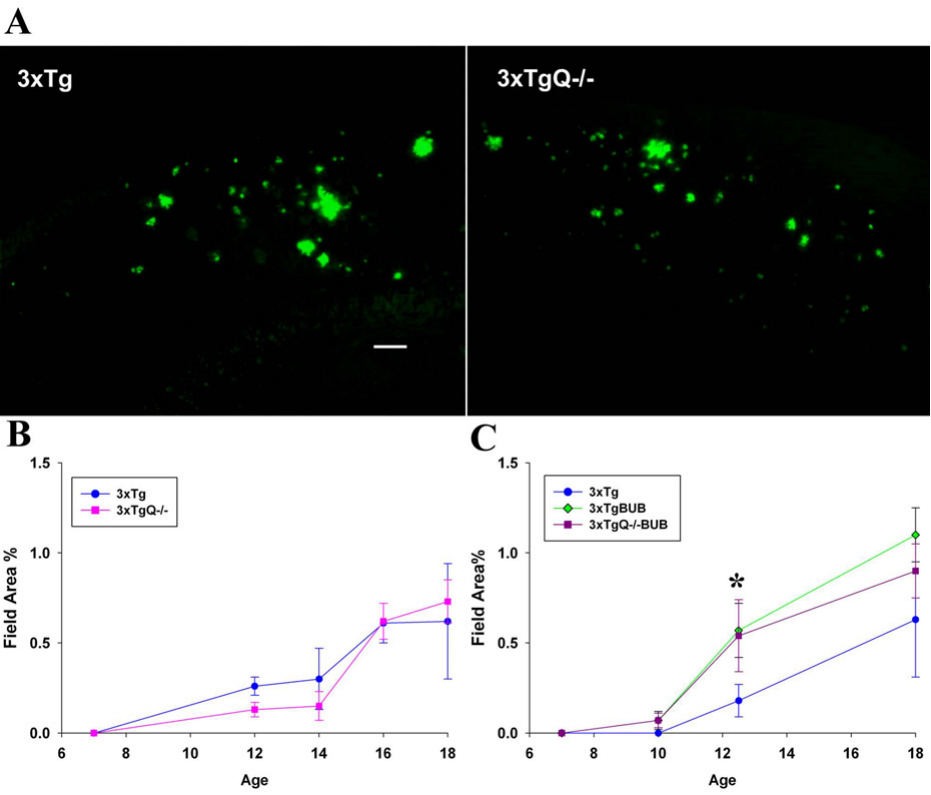
**Genetic deficiency of C1q does not alter fibrillar plaque pathology in 3xTg, but kinetics of plaque deposition is increased in both 3xTg and 3xTgQ-/- backcrossed to BUB**. A. Representative pictures of thioflavine staining in the subiculum area of 3xTg and 3xTgQ-/- at 16 m (scale bar: 100 um). B, C. Thioflavine quantification by image analysis in the subiculum area of the 3xTg and 3xTgQ-/- (B) or the 3xTg and 3xTgBUB and 3xTgQ-/-BUB (C) at different ages. Data points are the average of n animals +/- SE at different ages. (B) 3xTg, 3xTgQ-/- at 7 m n = 8,8; at 12 m n = 11,10; at 14 m n = 3,6; at 16 m n = 9,15; at 18 m n = 3,4. (C) 3xTg, 3xTgBUB, 3xTgQ-/-BUB at 7 m n = 3,3,5; at 10 m n = 8,3,8; at 12.5 m n = 7,7,8; 18 m n = 4,5,6. *p < 0.05 (3xTg vs 3xTgBUB) using ANOVA single factor.

The deposition of fibrillar plaques as detected by thioflavine staining (which only labels fibrillar amyloid) was assessed by image analysis in the subiculum area of the hippocampus (the area where pathology starts in the 3xTg model) at 7,12,14,16 and 18 m in the 3xTg vs. 3xTgQ-/- group. As shown in Figure 1A, and 1B, there is no significant difference between the 3xTg and 3xTgQ-/- in the amount of fibrillar plaques seen at any age. Similarly, total amyloid immunostaining using 6E10 antibody, did not show any differences between the 3xTg and 3xTgQ-/- groups (data not shown). When the same studies were performed using 3xTg and 3xTgQ-/- on BUB background, again no differences in the amount of plaques were seen at any of the ages studied (7,10,12.5 and 18 m) in the presence or absence of C1q (Figure 1C). However, the 3xTg on BUB background did have higher thioflavine staining than the 3xTg (mixed background) at 12.5 and 18 m with a 3 fold greater amount at 12.5 m that was statistically significant (p < 0.05).

**Figure 2:**
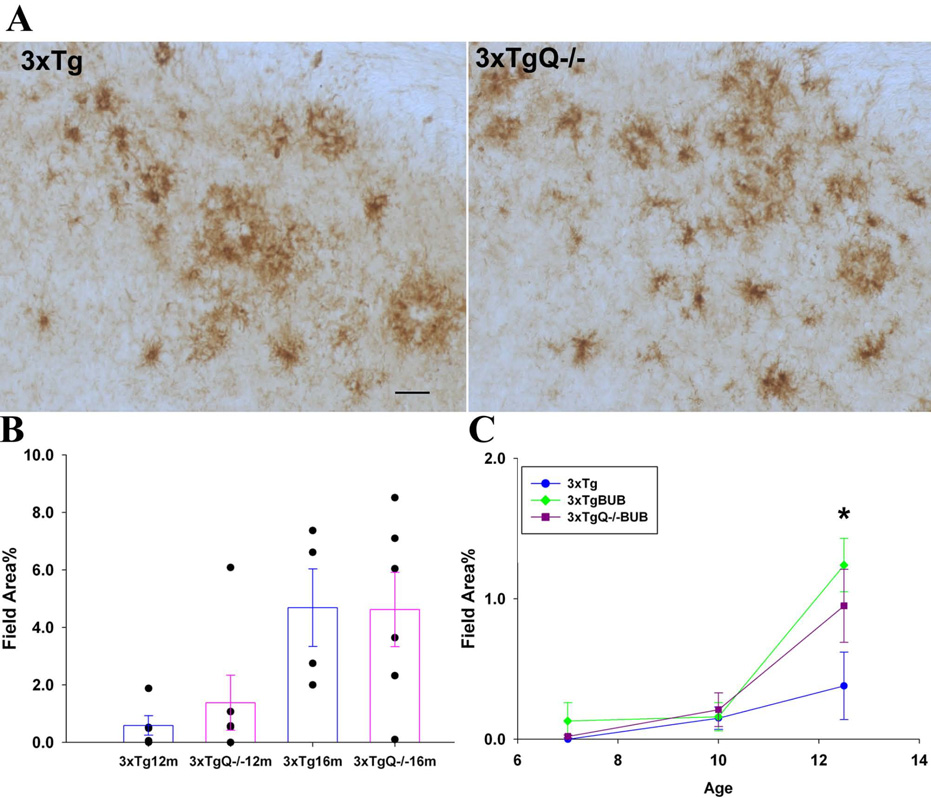
**Microglial reactivity associated with plaques is not altered by the absence of C1q in the 3xTg mice on either background, although 3xTgBUB mice show a statistically significant higher CD45 reactivity than 3xTg at 12.5 m**. A. CD45 immunostaining in the subiculum area of 16 m 3xTg or 3xTgQ-/- animals (Scale bar: 50 um). B. Image analysis of CD45 in representative trials at 12 and 16 m. Data points are individual animals. Bars are the average of n animals +/- SE. 12 m 3xTg n = 5, 3xTgQ-/- n = 6, 16 m 3xTg n = 4, 3xTgQ-/- n = 6. Data is representative from one of two different trials (total of at least 11 mice per genotype). C. Progression of the CD45 pathology in 3xTg, 3xTgBUB and 3xTgQ-/-BUB at different ages. Data points are the average of n animals +/- SE at ages noted. 3xTg, 3xTgBUB, 3xTgQ-/-BUB at 7 m n = 3,3,5; at 10 m n = 8,3,8; at 12.5 m n = 7,7,8; *p < 0.02 (3xTg vs 3xTgBUB) using ANOVA single factor.

In contrast to previous comparison of Tg2576 and Tg2576Q-/- mice [16], deletion of C1q did not affect the level of CD45 immunoreactivity (activated microglia) seen around the plaques in the 3xTg model (Figure 2A and 2B) or in the 3xTg model on BUB background (Figure 2C). However, as seen with the thioflavine results, a trend for an increase in the CD45 staining is observed in the 3xTgBUB at 12.5 m when compared with 3xTg, a difference that is statistically significant (p < 0.02). The variability in all pathological markers among the individual animals in all the groups (at all ages) was considerable (as can be seen by the SE) even among littermates, suggesting that it might be due to genetic factors. This variability was particularly evident in the microglia reactivity with some groups of animals displaying little or no clear increase of microglial reactivity (CD45, MAC-1 and Iba1 staining) in parallel with the increases seen in thioflavine plaques. However, astrocytes (labeled with GFAP) surrounding plaques were found in levels corresponding relatively to the thioflavine load (data not shown). The reason for this variable microglial reactivity observed in the 3xTg is currently unknown.

**Figure 3:**
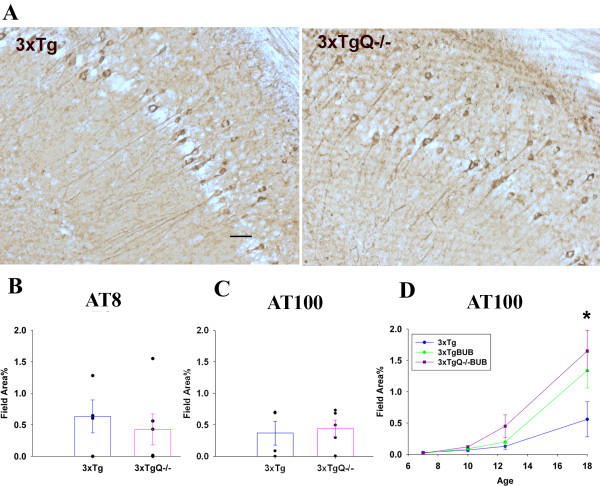
**Neurofibrillary tangles/phosphorylated tau pathology was similar in the 3xTg and 3xTgQ-/- mice, while a significant increase was seen in the 3xTg BUB**. A. AT8 immunostaining in the subiculum area of the 3xTg and 3xTgQ-/- mice at 16 m. Scale bar: 50 um. B, C. AT8 and AT100 quantification by image analysis in the subiculum of the 3xTg and 3xTgQ-/- mice at 16 m. AT8 n = 4,6, AT100 n = 4,5 (data is representative from two different trials for the AT100). Data points are individual animals. D. AT100 quantification at different ages in the 3xTg, 3xTgBUB and 3xTgQ-/-BUB. Points are the average of n animals +/- SE at different ages. 3xTg, 3xTgBUB, 3xTgQ-/-BUB at 7 m n = 3,3,5; at 10 m n = 8,3,8; at 12.5 m n = 7,7,8; at18 m n = 4,5,6. *p < 0.05 (3xTg vs 3xTgBUBQ-/-) using ANOVA single factor.

The 3xTg model not only develops plaque pathology but also accumulates neurofibrillary tangles that contain hyperphosphorylated tau. Immunostaining and image analysis of AT8 and AT100 (antibodies for phosphotau) showed no difference in the amount of phosphorylated Tau between 3xTg and 3xTgQ-/- (Figure 3A and 3B) or between 3xTgBUB and 3xTgQ-/-BUB (Figure 3C) at any age tested indicating that the lack of C1q did not alter the amount of tau phosphorylation and the progression of neurofibrillary tangles. However, importantly, there is a trend of an increase of AT100 reactivity in the 3xTg and the 3xTgQ-/- on the BUB background relative to the 3xTg which reached statistical significance at 18 m between the 3xTg vs 3xTgQ-/-BUB groups (p < 0.05).

### Evidence of classical pathway activation is not seen associated with plaques in brain of 3xTg or 3xTgBUB

**Figure 4:**
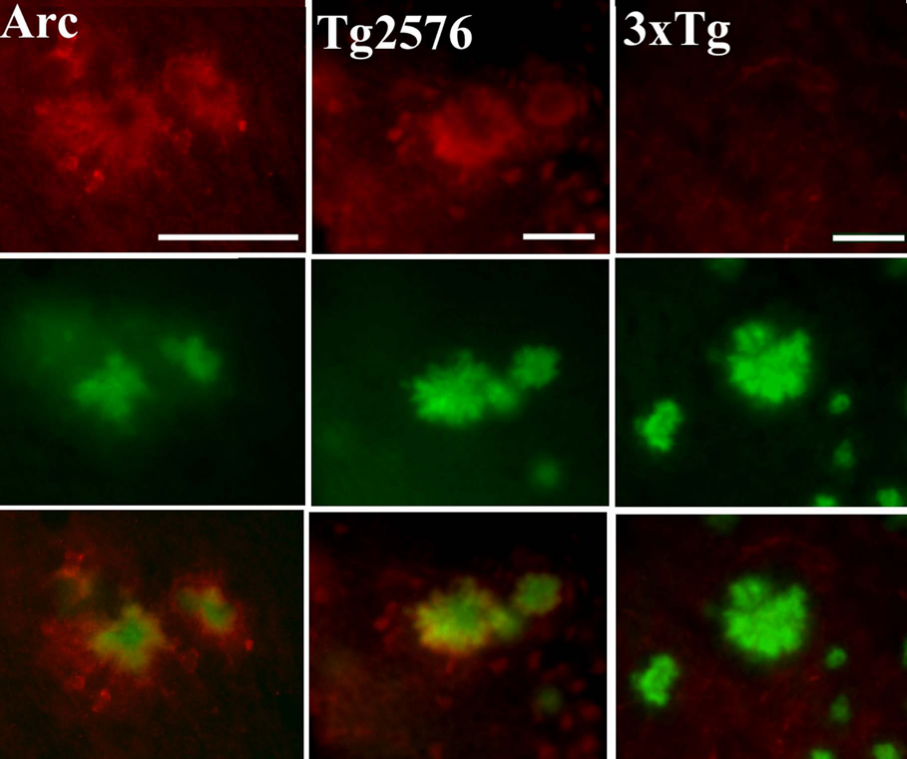
**C1q deposition is not detected on plaques in 3xTg in contrast to other APP mouse models**. Representative pictures of C1q (red) and thioflavine (green) and merge (bottom row) show colocalization in Arc48 mice at 9 m (left) and Tg2576 at 18 m (middle), with no detectable staining of C1q in 3xTg at 22 m (right). Scale bar: 50 um.

There is evidence that supports the hypothesis that complement activation by ß-amyloid fibrils occur in AD (Tenner and Fonseca, 2006), and that this activation might be, in part, responsible for the recruitment of activated glia and the generation of an inflammatory environment in the area of the plaque that can enhance neuropathology. In several transgenic models of AD complement factors have been shown associated with the plaques [31-33]. In the Arc48 model [26] which develops plaque pathology at an early age, C1q is associated with plaques as the first thioflavine plaques develop (around 2 m) and increases with age (data not shown), strongly colocalizing with thioflavine (Figure 4, left). In the Tg2576 model, C1q is also associated with thioflavine plaques (Figure 4, middle). Surprisingly, neither the 3xTg (Figure 4, right) nor the 3xTg on the BUB background (data not shown) showed detectable levels of C1q colocalizing with plaques.

**Figure 5:**
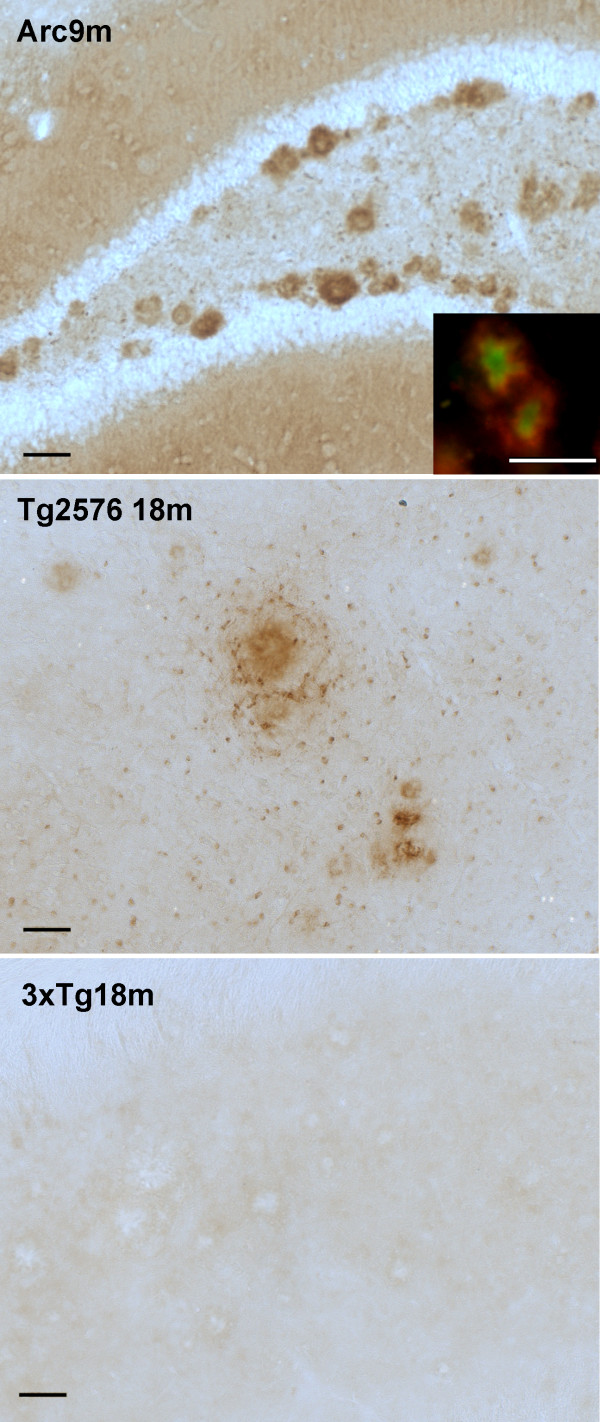
**C4 is highly expressed in the plaques of Arctic and Tg2576 models but absent in the 3xTg model**. C4 immunostaining (brown) in the Arc48 mouse hippocampus at 9 m (top) and Tg2576 cortex at 18 m (middle), and 3xTg subiculum at 18 m (bottom). Colocalization of C4 (red) with thioflavine (green) (top inset) in Arc48 model. Scale bar: 50 um.

C4 immunostaining with an antibody that recognizes C4 and its cleavage fragments C4b and C4d showed that C4 is present in the Arc48 model (Figure 5, top) and colocalizes with thioflavine (Figure 5, top inset). As previously reported [33], C4 is deposited on plaques in the Tg2576 mice and also expressed in oligodendrocytes (Figure 5, middle) as early as 12 m, while undetectable in the Tg2576Q-/- mice of the same age. In contrast, no C4 was detected on the plaques of the 3xTg model even at advanced ages (Figure 5, bottom) or in the BUB (high complement hemolytic activity) strain (data not shown).

### Expression of C3 and cleaved C3 fragments in the 3xTg brain compared to other APP transgenic models

**Figure 6:**
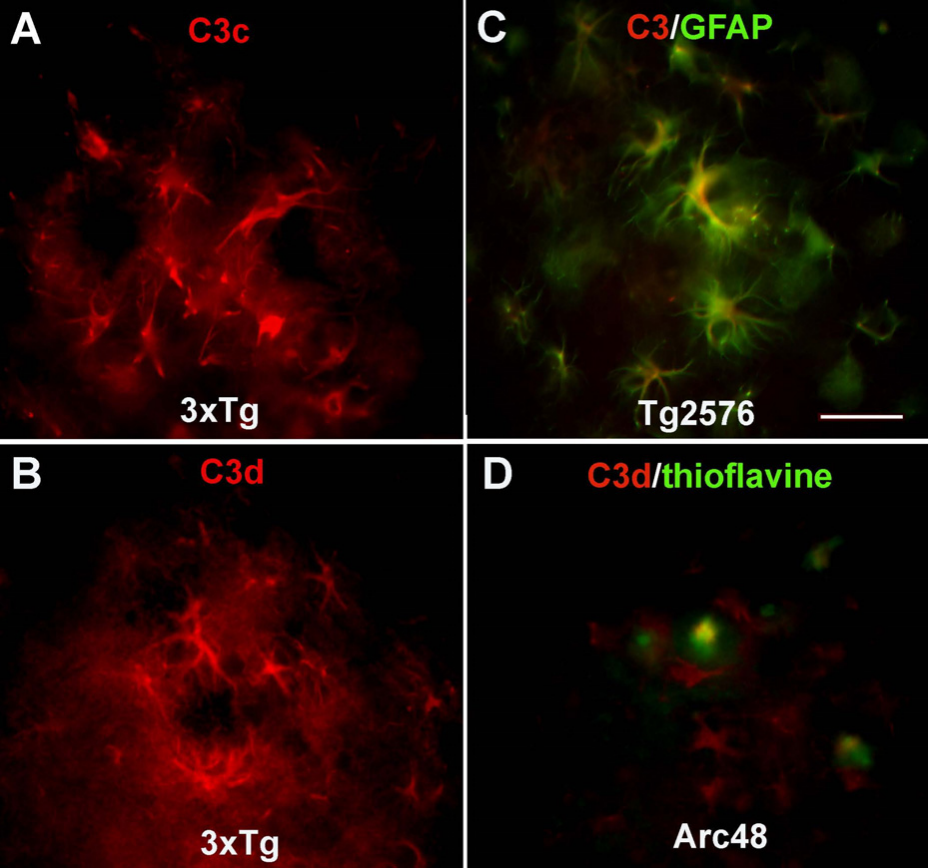
**C3 is expressed in astrocytes clustered around plaques in the 3xTg similarly to other APP models of AD, but is not associated with plaques as shown in the Arc48 model**. C3 immunostaining using anti human C3/C3c (A) and anti human C3/C3d (B) (red) in astrocytes clustered around plaques in the subiculum of the 3xTg mice at 18 and 22 m respectively. C. Colocalization (merged image) of anti mouse C3 (clone11H9) (red) with GFAP (green) in Tg2576 15 m. D. Colocalization of anti human C3/C3d (red) with thioflavine (green) in the Arc48 model at 9 m. Merged images shows that this anti C3/C3d antibody labels astrocytes (red) and also colocalizes with thioflavine plaques (yellow). Scale bar: 50 um.

C3 immunoreactivity was tested using two monoclonal antibodies, the anti mouse C3 antibody (Clone 11H9) that recognizes both intact C3 and the cleaved fragments C3b, iC3b and C3d while anti mouse C3b/iC3b/C3c (clone 2/11) is specific only for the cleaved [34] fragments and two polyclonal anti human C3 antibodies that react with either the C3c (C3/C3c antibody) or the C3d (C3/C3d antibody) in the native or cleaved molecule. Results of immunohistochemical analysis are summarized in Table 1. Reactivity was detected in subpopulations of astrocytes associated with plaques, shown by the polyclonal anti human C3/C3c in 3xTg (Figure 6A), Tg2576 and Arc48 (data not shown), and the monoclonal anti mouse C3 (clone 11H9) in the Tg2576 (Figure 6C) and 3xTg (data not shown), consistent with the known synthesis of C3 by astrocytes in an injured or inflammatory environment. In the Arc48 mice at younger ages the 11H9 antibody stained only astrocytes, but at later ages (13 m) plaques were also labeled (data not shown). While staining astrocytes less prominently, the anti human C3/C3d polyclonal antibody also showed reactivity associated with the plaques in the Arc48 model at older ages (9 m, Figure 6D), suggesting the presence of the activation fragment of C3 since C3d contains the thioester that can form covalent binding with an activator (here the fibrillar amyloid). In contrast, while the polyclonal anti human C3/C3d antibodies did label astrocytes surrounding plaques in the Tg2576 (data not shown) and 3xTg (Figure 6B) similar to the Arc48, the association of C3d on plaques was not detected in either Tg2576 (data not shown) or in the 3xTg with this antibody (Figure 6B). The monoclonal 2/11 which reacts with activated/cleaved C3 only (C3b/iC3b/C3c), stained plaques in Arc48 (13 m) (data not shown) and Tg2576 [33] although no reactivity was detected in 3xTg, further demonstrating differential plaque associated complement components. The 3xTgBUB strain reacted similarly to the 3xTg with all anti C3 antibodies (data not shown).

**Table 1:** Summary of immunoreactivity observed with anti C3 antibodies

	Anti mouse C3 (clone 11H9)	Anti human C3/C3c	Anti human C3/C3d	Anti mouse C3b/iC3b/C3c (Clone 2/11)
**3xTg**	A^1^	A^2^	A^3^	negative

**Tg2576**	A^4^	A	A	P

**Arc 48**	A/P	A	A/P^5^	P

^1 ^A, Astrocytes; P, Plaques; ^2 ^Figure 6A, ^3 ^Figure 6B, ^4 ^Figure 6C, ^5 ^Figure 6D

### Properdin is associated with plaques in APP transgenic mice

**Figure 7:**
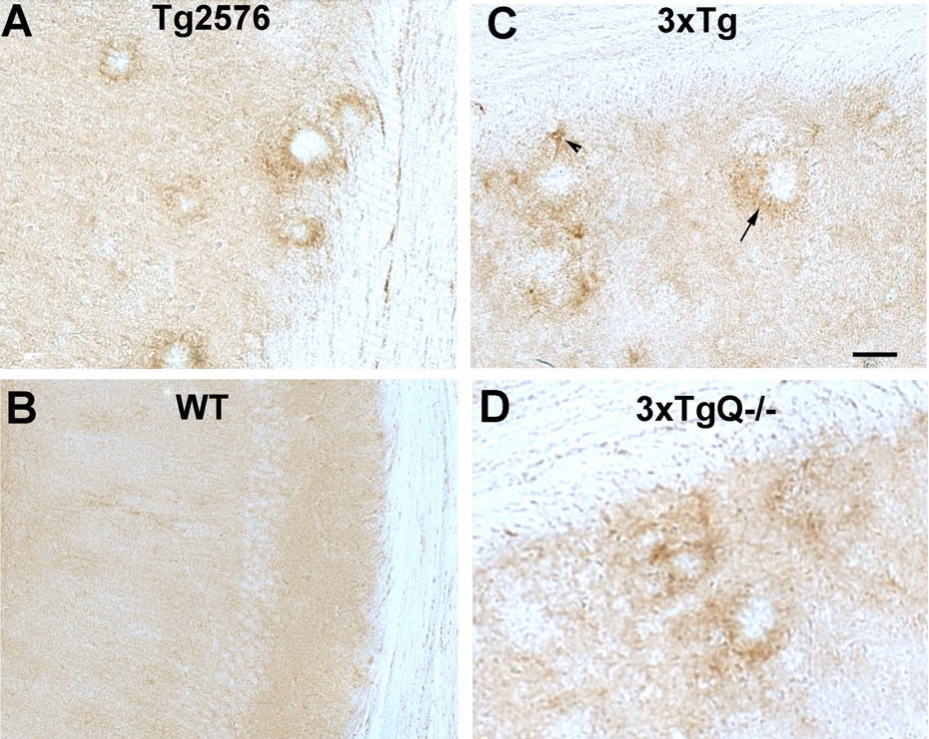
**Properdin is associated with plaques in Tg2576 and 3xTg models**. Representative pictures of properdin immunostaining using anti mouse properdin antibody in the hippocampus of 17 mo Tg2576 (A) and 17 mo wild type littermate (B), and the subiculum of 16 mo 3xTg (C) and 18 mo 3xTgQ-/- (D). Arrow shows properdin associated with plaques and arrowhead points to occasional astrocyte staining. Scale bar: 50 um.

Since activated C3 was not detected on plaques in the 3xTg mice with the antibodies available but a previous report had demonstrated a 50-70% decrease of pathology following treatment with a C5aR antagonist [17], we looked for the presence of properdin, a positive regulator of the alternative pathway, as evidence of alternative pathway activation in these mice. Using an anti mouse properdin antibody [28], properdin reactivity was clearly present associated with plaques (with occasional astrocyte staining) in the Tg2576 (Figure 7A), 3xTg (Figure 7C), as well as 3xTgBUB and Arc 48 (data not shown) models. Properdin staining was also observed in 3xTgQ-/- (Figure 7D) and 3xTgQ-/-BUB brain (data not shown). No staining was detected in nontransgenic littermates or controls (Figure 7B). These data are consistent with alternative pathway activation in the 3xTg as well as Tg2576 and Arc mouse models.

### Similar increases in inflammatory gene expression are detected in the 3xTg Q-/-BUB as in 3xTgBUB

**Figure 8:**
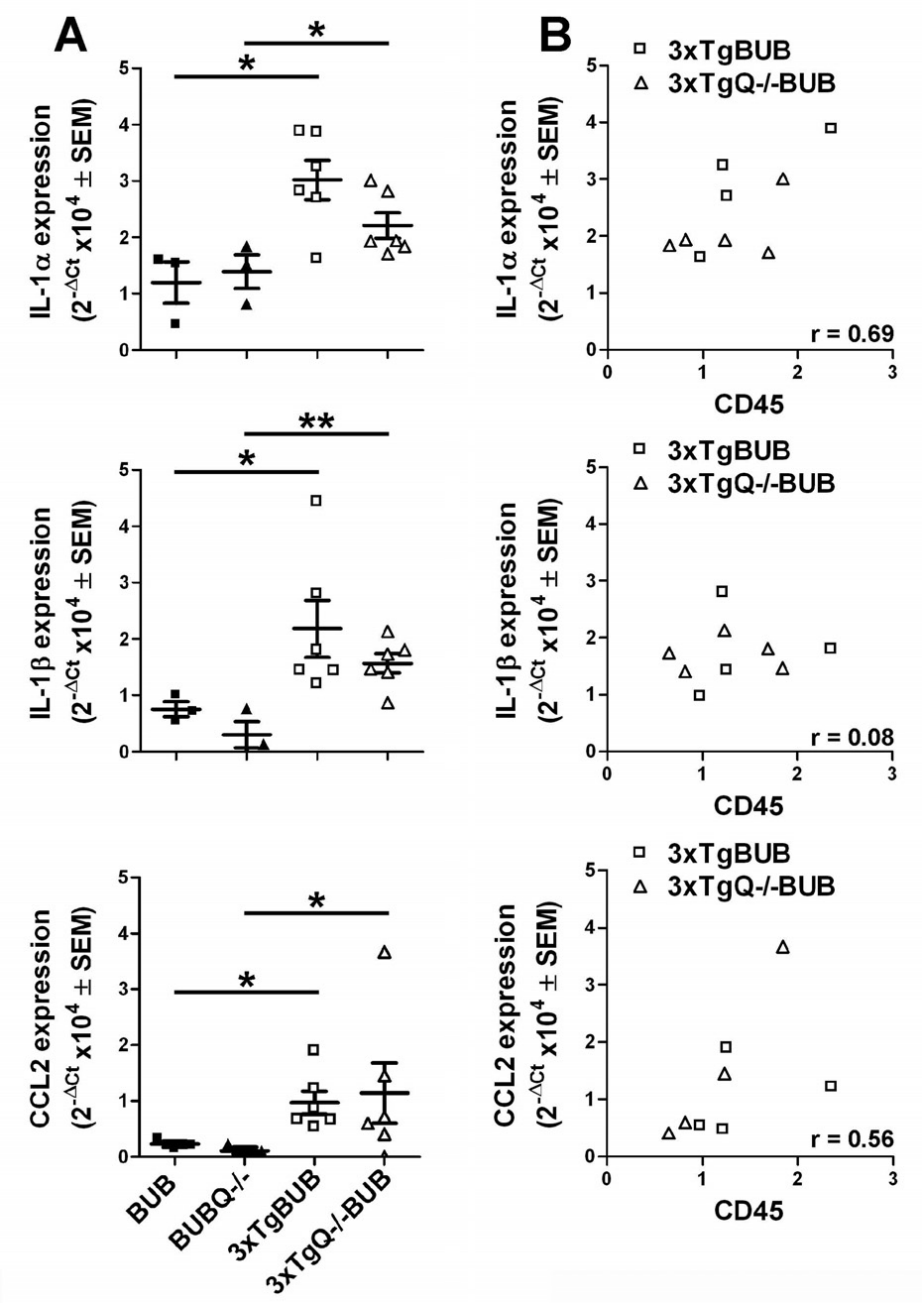
**Similar increased expression of the proinflammatory markers IL-1α, IL-1β, CCL2 and CD45 in 3xTgBUB and 3xTgQ-/-BUB mice compared to non-transgenic BUB mice**. A. Gene expression of IL-1α, IL-1β and CCL2 in the hippocampus of 18 m old 3xTgBUB and 3xTgQ-/-BUB mice compared to the non-transgenic BUB and BUBQ-/-mice was assessed by qRT-PCR. Results are represented as individual animal scatter dot plots with mean ± SEM (BUB and BUBQ-/-, n = 3 and 3xTgBUB and 3xTgQ-/-BUB, n = 6) of fold-difference (2^-ΔCt^). B. Increases in IL-1α are positively correlated with increased CD45 (r = Pearson's correlation coefficient)*, p < 0.05 and **, p < 0.01.

Expression of inflammatory gene transcripts in cortex and hippocampus of 18 m old 3xTgBUB, 3xTgQ-/-BUB and nontransgenic BUB and BUBQ-/- controls was assessed by qRT-PCR. (Figure 8) Clearly detectable increases in IL-1α, Il-1ß and CCL2 (MCP-1) transcripts were detected in the hippocampus of the 3xTgBUB relative to the nontransgenic BUB mice (Figure 8A). Specifically, for IL-1α and IL-1ß, a 3 fold-increase, (p = 0.014 and 0.048, respectively) in gene expression was detected in the 3xTgBUB vs the BUB nontransgenic mice, and a 2- and 5-fold increase was seen (p = 0.042 and 0.0038, respectively) comparing 3xTgQ-/-BUB to the BUBQ-/-. For CCL2 there was a 4-fold increase in transcript levels in the 3xTgBUB relative to the BUB (p = 0.046) and a 12-fold increase comparing the 3xTgQ-/-BUB to the BUBQ-/- (p = 0.035). However, there are no statistically significant differences between the C1q knock out and C1q sufficient animals (Figure 8A) in agreement with the pathology results. No such differences were detected in the cortex (data not shown), correlating with the little to no pathology in the cortex of these mice at this age (pathology is predominantly in the subiculum area of the hippocampus in these animals). No significant differences in IL-6 or TNFα mRNA levels were identified in either region of the brain (data not shown) in any of the animals tested. Comparison of IL-1α, Il-1ß and CCL2 transcript expression with the level of immunohistochemical CD45 reactivity (a general marker of microglial reactivity) showed a trend toward a positive correlation with the expression of these elevated transcripts, although this reached significance only in the case of IL-1α (p = 0.038, Figure 8B).

## Discussion

The presence of complement components and indicators of inflammation in AD brain suggests that complement activation may contribute to the progression of AD, and if so, can be a novel therapeutic target. *In vitro *data, demonstrating the ability of fibrillar ß-amyloid to activate both the classical and alternative pathway is consistent with complement pathway activation by fibrillar amyloid plaques *in vivo *[12,13]. In our previous publication, treatment of 3xTg mice with a C5a receptor antagonist [C5a is a proinflammatory peptide that can be generated as a result of complement activation] showed marked decreases in both amyloid plaque and hyperphosphorylated tau pathology [17]. In the present study the contribution of complement to pathology was defined in the 3xTg model. To eliminate classical complement pathway activation, mice were first bred to generate C1q-deficient 3xTg mice. Conversely, to enhance complement activity, 3xTg were backcrossed 6 generations to the BUB background, a strain with higher levels of serum complement hemolytic activity. No differences in thioflavine plaques, reactive glia, hyperphosphorylated tau, or selected inflammatory markers were detected between the C1q -sufficient and -deficient 3xTg mice on either background, which is in contrast to previous studies with Tg2576 lacking C1q that had shown reduced gliosis and increased neuronal integrity [16]. Consistent with the lack of C1q-dependent pathology in the 3xTg, there were no early components of the classical complement pathway associated with the plaques, again in sharp contrast to the Tg2576 [16] and the Arc48 model (Figure 5). Therefore, the positive effect of the specific antagonist for the C5aR (PMX205) in suppressing pathology in the 3xTg animals [17] suggests that either alternative pathway of complement activation is the predominant and sufficient mechanism of C5a-generation in this animal model (as the alternative pathway is independent of C1q), that the newly described C3-independent enzymatic cleavage of C5 [35] may be the source of the C5a contributing to pathology in these mice or that the protective effect of PMX205 is independent of C5a effects.

The presence of properdin, an alternative complement pathway component that can stabilize the C3 convertase as well as initiate alternative pathway activation [36], associated with amyloid plaques in 3xTg as well as 3xTgQ-/- animals supports the possibility that the alternative pathway is activated in this AD model. However, an additional contribution of the C3-independent generation of C5a cannot be ruled out at this point.

A role for the C5a activation fragment of complement in many inflammatory disorders including neurodegeneration has been well documented (reviewed in [37]). The abrogation of AD pathology in the 3xTg model backcrossed to the C5 deficient FVB strain (D.A.Morrissette, PhD dissertation, 2009, UCI) as well as the delay in amyloid accumulation in the C5-deficent mice containing the human APP gene under its own promoter [38] supports, though does not prove, a contribution of C5a. While it is possible that PMX205 is inhibiting other receptors, PMX53, the close homolog of PMX205, was screened for inhibition of 44 different receptors including 4 ion channels and two transporter proteins and shown to exert inhibition with only 4 other receptors and only at a minimum of 3 fold higher concentration than that which inhibits CD88/C5aR [39]. Treatment with PMX205 in mice had no effect on dampening leukocyte migration to the CNS in response to intracranial inoculation with a neurotrophic coronavirus (T.E.Lane, UC, Irvine, personal communication) [17], emphasizing the variety of selective chemotactic and inflammation inducing mechanisms available that are not inhibited by this antagonist.

A second major finding of this study is that thioflavine positive plaques and glial accumulation was detected earlier (by approximately 2 months) in the 3xTgBUB mice relative to the 3xTg (Figure 1 and 2), consistent with the possible greater generation of detrimental C5a in the BUB background. Since there was no evidence of C1q and C4b associated with plaques in the 3xTg BUB, similar to the mixed background 3xTg, greater alternative pathway complement activation or greater C3-independent C5 cleavage (both of which can lead to higher C5a generation) in BUB may be the basis for this accelerated pathology. Although C3b stably bound to plaques was not detected in the 3xTg, it is possible that low levels of alternative pathway activation could occur, but that amplification is highly regulated (such as by the complement regulatory protein Crry previously shown to be present at high levels in mice [32]) resulting in very low surface bound C3b that may be difficult to detect by any of antibodies used.

These data, and other results from studies by us and others, suggest that the role of complement in AD is complex, with evidence for both detrimental and beneficial functions [16,17,40-42]. For example, transgenic over expression of the murine complement inhibitor of C3 (Crry) or generation of a C3 deficient APP mice resulted in enhanced pathology in these mouse models suggesting a protective contribution of complement [41,43] (possibly due to the opsonic effect of C3b for amyloid or cellular debris that is missing when classical and alternative pathways are blocked by C3 inhibition or deletion). This protective role is also consistent with the recent report demonstrating a correlation between induction of early components of complement and suppression of Aß deposition in the TgCRND8 AD mouse model [44]. However, deletion of C1q in the Tg2576 and APPPS1 models of AD supported a detrimental role for complement activation since the Tg2576C1q-/- and APPPS1C1q-/- mice showed less reactive glia surrounding plaques and increased synaptophysin than the Tg2576 or APPPS1 [16]. The protection given by the lack of C1q (and thus lack of the classical pathway for complement activation) was substantial but not complete, suggesting that the alternative pathway and/or other non complement mediated events contribute to the inflammatory reaction around the plaques. The deposition of C3b on the plaques of Tg2576C1q-/- in the absence of C1q and C4 demonstrated that the alternative pathway is activated in the Tg2576C1q-/- mice [33]. The presence of properdin on plaques of all models assessed here indicates that either or both complement pathways are activated in AD mice.

The 3xTg is the first transgenic model of AD in which early components of classical (C1q and C4) complement pathway have not been detected associated with thioflavine positive plaques. Robust deposition of C1q and C4 in the Tg2576 mice [33] and the APP23 mice [32] or C1q in the APP/PS1 [31] on plaques has been demonstrated. In addition, the mouse model for cerebral microvascular amyloid showed increases in C1q, C3 and C4 in areas with fibrillar amyloid deposits [45]. We cannot rule out the possibility that there is transit binding of C1q in C1 (and thus limited activation of the classical complement pathway) on the plaques of the 3xTg or increased plaque binding of complement regulators such as C4BP (known to bind to plaques in AD brain) [46]. In any case, the possibility that the complement pathways are more robust in the Tg2576, APP23, (and APP/PS1) provides a plausible explanation as to why the progression of the pathology is faster in those strains than in the 3xTg. Similarly, the accelerated progression of pathology in the 3xTg on BUB background is consistent with a role for complement in determining the rate of progression of the disease. The different C3 and C5 convertases involved in the classical and alternative pathways and/or the balance of both pathways might contribute differently to the extent of downstream activation (C5a generation) and to the resulting pathology in these models. The potential polymorphism in CR1 associated with human AD also suggests a point of control of complement activation, since CR1 is a critical regulator of C3 convertase activity in humans, and remains to be further investigated.

Interpretation of the immunohistochemical data assessing the presence of C3 in inflammatory disease models requires an understanding of the various forms of C3 being recognized by the antibodies used. While C3 has been shown to be synthesized by astrocytes and microglia in culture [47] and in brain tissue by in situ hybridization in neurodegenerative diseases or with injury or inflammation [7,48], reports of the association of both native and activated C3 differ among mouse models of neurodegeneration. For example, plaque labeling at different levels, but not astrocyte labeling was observed in the APP23 model with the polyclonal anti C3d antibody [32,49], while weak to absent plaque staining in Tg2576 mice was reported with this antibody [49]. Here, the polyclonal anti human C3/C3d antibody labels astrocytes (probably via epitopes on C3d exposed in intact C3) in the Arc48 model and in the Tg2576 and 3xTg. In addition, the monoclonal anti C3 antibody, clone 11H9 (that recognizes intact C3), labeled only astrocytes in 3xTg and Tg2576 mice, suggesting that this C3 was newly synthesized, uncleaved C3 (consistent with the identification of C3 as an acute phase protein) [50]. The polyclonal C3/C3c antibody that recognizes the C3c region within C3 (C3c can also be dissociated from the thioester surface bound C3d upon a second cleavage of C3b by Factor I), labeled mainly astrocytes in all three models. The lack of astrocyte labeling by the 2/11 anti C3b/iC3b/C3c that recognizes only cleaved C3, supports the conclusion that the astrocyte labeling in these models represents native, uncleaved C3. Importantly, activated C3 neoepitopes detected by the 2/11 monoclonal antibody were associated with plaques in the Arc48 model but not on the plaques in the 3xTg. Plaques in Arc48 were also stained with the polyclonal anti C3/C3d antibody likely reflecting reactivity with activated C3b cleaved to C3d on those plaques. Overall, these results are consistent with differential activation of C3 in these mouse models. [It should be noted, while we consistently observed astrocytes labeling with all the C3 antibodies tested (except 2/11, which is specific for cleaved C3) in all three mouse models, in another murine model of neurodegeneration C3 was reported exclusively in microglia [45].]

In conclusion, there is substantial evidence showing C1q, C4 and C3 strongly associated with fibrillar plaques in human AD brain and Down's Syndrome with AD [3,5,51,52] even in early stages of the disease correlating with the appearance of fibrillar amyloid [2]. Complement proteins of the alternative pathway are also detected associated with plaques in the human disease [53]. The production of complement factors by brain cells [6,7,54] and the presence of the terminal complement membranolytic complex C5b-9 detected on plaques and tangles in human AD [9] provide further evidence that complement is present and fully activated in AD brain and therefore might contribute to the enhancement of neurodegeneration at later stages of the disease when fibrillar plaques are present. Mouse models of AD exhibit to some degree many of the pathological features of AD [55,56]. However, the absence of definitive evidence for some late complement factors [32,49] and/or differences in the ratios of complement components and complement inhibitors between AD and AD animal models have been reported [32] suggesting that there might not be quantitatively comparable complement activation in mouse models as in human AD. Here, the presence of properdin in all mouse models of AD provides evidence for the activation of the alternative complement pathway, consistent with the benefit demonstrated in the 3xTg as well as Tg2576 mice of treatment with an antagonist of the proinflammatory C5a receptor [17]. Nevertheless, the development of new mouse models that more closely mimic the human system in all aspects of the disease will further improve the ability to assess the contribution of complement to AD neuropathology, define the targets most likely to promote beneficial effects and/or prevent detrimental activities [57-59] and aid in developing treatments for this devastating human disease.

## Competing interests

The authors declare that they have no competing interests.

## Authors' contributions

MIF carried out part of the IHC experimental work and statistical analysis, prepared the figures and drafted the manuscript. SHC generated and genotyped the animals used in the study, processed the tissue, and performed some of the IHC experiments and statistical analysis. AB processed some of the tissue used and performed some IHC experiments and contributed to the analysis. YK provided the anti properdin antibody, and design of some IHC experiments. MEB designed and analyzed the qPCR experiments and contributed to the manuscript, and DGP performed qPCR experiments and contributed to the analysis. AJT designed the study, contributed to the analysis of all data and to the preparation of the manuscript. All authors read and approved the final manuscript.
